# Working with democratically elected councillors: Reflections on research engagement in local government

**DOI:** 10.1016/j.puhip.2025.100701

**Published:** 2025-12-11

**Authors:** Andrew Passey, E.M. Brown, James Woodall

**Affiliations:** a School of Health, Leeds Beckett University, United Kingdom and Health Determinants Research Collaboration Wakefield, United Kingdom; b Health Determinants Research Collaboration Wakefield, Wakefield Council, United Kingdom

**Keywords:** Local councillors, Research engagement, Local government, Health Determinants Research Collaborations (HDRCs)

## Abstract

**Objectives:**

Although local politicians (councillors) in England are key decision-makers in relation to local services, little is known about how they use evidence in making decisions pertaining to the wider determinants of health. The setting-up of 30 Health Determinants Research Collaborations (HDRCs), commissioned to increase the capability and capacity of local authorities to use research and other evidence, provides an opportunity to plug this knowledge gap.

**Study design:**

A qualitative reflection on our initial experiences of working with councillors in one HDRC.

**Methods:**

We critically reflect on these interactions to extract wider learning that will be of interest to researchers and practitioners in local authorities and potentially research funders. We develop a continuum of interactions with councillors in the HDRC based on our experiences.

**Results:**

Organisational positionality plays a crucial role in shaping research interactions and outcomes. By focusing on existing practices, preferences, and strengths, researchers can move beyond the deficit models that often dominate academic discourse. Engaging with councillors is rarely a linear process; it demands parallel approaches and a high degree of adaptability. Researchers must also remain attentive to both the challenges and opportunities presented by organisational structures and electoral cycles. In this context, mixed-methods research demonstrates resilience, accommodating the varied levels of engagement and involvement among councillors.

**Conclusions:**

We conclude from these experiences that research with councillors and building their capability to use research evidence in their work requires sustained, flexible, and relational approaches that are responsive to the political and organisational realities of local government.

## 1 Introduction

English local authorities have responsibilities relating to wider determinants of health [1]. Local politicians (councillors) are ‘primary decision makers in determining the direction of local services’ [2], but little is known about how they use evidence of different types in making decisions. This contrasts with evidence about how civil servants and central government policymakers use evidence in their work [3]. The formation of 30 Health Determinants Research Collaborations (HDRCs),^1^, ^2^ commissioned to increase the capability and capacity of local authorities to use research and other evidence, provides an opportunity to plug this knowledge gap and develop interventions to enhance evidence use. Our HDRC comprises the council, two universities and local voluntary sector partners. One councillor was involved in the bid to the funder.

Studies suggest councillors employ many evidence types and sources, but little directly from academia (see for example [4]). Councillors are advocates for their constituents and local areas [5], as well as for their political parties and priorities. Local public preferences are important to councillors and as such influence policy formation and implementation [6]. Many councils have a Leader and Cabinet system,^2^ in which senior councillors are responsible for making many decisions on behalf of the council. Councillors might be members of scrutiny committees that review council policies, decisions and performance - others will be backbenchers [7]. They face significant calls on their time, and time scarcity presents a significant constraint on engaging them with and in research [8]. Research has highlighted deficit discourses that point to a lack of necessary knowledge, skills sets and motivations among councillors as one reason for performance ‘failures’ in local authorities [9], which we were mindful of not reproducing.

## 2 Methods

Initial work with councillors involved foundational mixed-method research to understand and assess their current use of research and evidence. This was required to increase our understanding about councillors' views, experiences and preferences, findings that will underpin development of products to support evidence use as part of the HDRC's core work. This paper involves qualitative reflections of our experiences of working with councillors on this programme as an example of ‘learning by doing’ [10], rather than reporting empirical findings from the research. The authors come from the council and academia and work in the HDRC. We shared reflections, identified common issues in our experiences, and developed a continuum of our work with councillors. This paper synthesises these critical reflections to extract wider learning that will be of interest to researchers, local authority practitioners and research funders.

## 3 Results: Reflections on working with councillors

Our continuum captures increased intensity of engagement with councillors (accessing, informing, engaging, involving). Our experience of moving along (and around) this continuum was not linear but instead included moments where we looped back to previous activities or within individual activities. These adaptations proved essential in navigating a political environment that does not lend itself to exclusively technocratic or linear approaches.

Initially, our HDRC was in the Corporate Centre of the council, along with the central policy function, which helped us *access* knowledge of how the council works and who to approach in the first place. The programme's Senior Responsible Officers (SRO) sit on the council's corporate management team, ensuring strong prior relationships with councillors and the most senior officers in the council, which helped all stages of engagement. Our positionality allowed us to consult the council's Committee/Democratic Services team, which leads on day-to-day contact with councillors, before beginning our work. They advised contacting councillors directly to start building rapport, which opened this avenue of *access* to us.

Taking this advice and following endorsement from the HDRC advisory group and senior Council leaders, we *informed* councillors about the HDRC in a short email communication from the team lead and *engaged* a senior councillor (Cabinet member) as an advocate for us. Organisational support manifested in the SRO advocating for the HDRC and our proposed research at forums of senior councillors and officers. Relational support (trust from these senior officers) enabled us to *engage* councillors, including getting practical advice from them to help maximise the amount of councillor experiences of using evidence we could collect: interviews might work better than surveys, the importance of links between councillors and council staff, and advice on our sampling strategy, interview topic guide and survey questionnaire. We gained trust from being physically located with officers who themselves had strong relationships with other key officers and councillors, in effect being seen to be part of that wider team.

We planned to *involve* a sample of councillors as research interviewees. To do so, we first *engaged* councillors through inviting them to in-person briefing events hosted by our advocate councillor. The Member Development team advised us to hold these in person as, although attendance may be lower, engagement would be higher. All councillors were invited. Around 30 % attended, considered a positive level of attendance compared with other briefing events. Our objectives were to *inform* councillors about the HDRC in more detail, specifically around our proposed work with them and to *engage* them in discussion about what they had heard. We were also interested in any expressions of potential interest in being *involved* as research participants. This flexible approach reflects the real-world experience of gaining the attention of a cohort of often time-poor people. Attendees provided feedback on awareness levels among councillors and valuable lessons in how we might communicate with them. They highlighted how a Cabinet system limited the role of most councillors in decision-making activities, which meant we needed to adopt a broader perspective on councillors' activities. They also expressed reticence about the language of ‘health determinants’, preferring building blocks of health [11].

We maintained efforts at *involving* councillors as interviewees, including a senior officer prompting councillors on our behalf. This wave of *involving* proved successful, moving from three completed interviews up to that point to 11 three months later - 17 % of all councillors. *Involving* efforts also included trialling a draft survey instrument on councillors’ use of evidence with two councillors (one Cabinet member, one backbencher). Accordingly, we made some of the response categories clearer, but subsequent work to *involve* all councillors through the survey proved less successful. Response was below 10 %, despite us routing the request through political party managers or directly to independent councillors, as recommended by Committee Services. The survey had one significant outcome - two councillors who completed it expressed interest in remaining involved in follow-up work. Across these different activities we directly interacted with 28 councillors (approximately 45 % of the total).

## 4 Discussion: Wider learning

We suggest several wider learning points for working with councillors to build research capability and capacity in local government. First, positionality matters in structuring space to work with councillors. In our case, the HDRC was located centrally in the council, which provided ready access to corporate support and wide reach across the authority, as well as strong relationships between officers and between them and councillors. This is not a claim that being centrally located is any ‘better or worse’ than being in a Public Health team, but instead that positionality has influenced our experiences to date. Also, having a university researcher embedded in the council helped to break down relational barriers and build trust. Second, designing our research to access everyday experiences and perspectives of councillors helped us to focus on existing practices, preferences and strengths, rather than assuming a deficit model. Third, while ideally we may have changed the sequences by completing the *informing* and *engaging* activities before trying to *involve* councillors in the research, we only had limited moments when we could capture the attention of councillors, which meant we deliberately sought to make the most of those opportunities. Fourth is to be aware of organisational and electoral cycles, which might provide challenges (such as a budget-setting full Council meeting in the middle of our work that consumed much councillor attention) or opportunities (there were no planned elections in our council area in 2025, which enhanced the chance of councillors engaging with our research). Finally, our mixed-method approach meant that lack of involvement in the survey was compensated for by the interviews, which generated rich qualitative data. This will underpin evidence-informed support to councillors in using evidence in their decision-making, scrutiny and constituent case work.

Our experiences are drawn from foundational research with councillors to understand their existing use of evidence, as part of a wider programme designed to build their research capability. This work requires sustained, flexible, and relational approaches that are responsive to the political and organisational realities of local government. Councillors play important roles as critical decision-makers, by representing their constituents and by holding those who make decisions to account, yet our understanding of how they engage with evidence requires further exploration. Our intention here was to share our learning to inform others working in this space and to foster wider debate on how academia and local government can work synergistically to improve community and population health and well-being.

## Author statements

AP and JW conceived the idea for the paper, AP and EB developed the continuum. AP led drafting of the paper, with detailed comments from EM and JW. All authors approved the final version.

## Ethical statement

Ethical approval was not required given that the paper presents the reflections of the authors.

## Funding

Funding was provided by 10.13039/501100000272National Institute for Health and Care Research, NIHR158707.

## Declaration of competing interest

The authors declare that they have no known competing financial interests or personal relationships that could have appeared to influence the work reported in this paper.

## Footnotes

1 Health determinants research collaborations | NIHR.

2 How are decisions made? | Local Government Association.
